# How much heating is associated with magnetic resonance imaging in patients with pacemakers and implantable cardioverter defibrillators?

**DOI:** 10.1186/1532-429X-16-S1-P374

**Published:** 2014-01-16

**Authors:** Steven Mason, Jon-David Ethington, Raymond O McCubrey, Allison E Tonkin, Jeffrey L Anderson

**Affiliations:** 1 Intermountain Heart Institute, Intermountain Medical Center, Murray, Utah, USA; 2 Division of Cardiology, University of Utah School of Medicine, Salt Lake City, Utah, USA

## Background

Background: Magnetic Resonance Imaging (MRI) provides unique diagnostic information yet is contraindicated with conventional pacemakers (PMs) and implantable cardioverter defibrillators (ICDs). MRI with careful monitoring is feasible with PMs/ICDs, but safety continues to be questioned, including heating of device components.

## Methods

Methods: We tested the effect of MRI on local tissue heating over PM/ICD pulse generators (PGs) in a consecutive series of 34 non-thoracic scans in 30 patients (pts) enrolled in an ongoing registry study. MRI was performed with a General Electric 1.5 tesla model Signa HDXT scanner. Temperatures were taken immediately pre- and post-study with an infrared thermometer beamed at the center of the PG pocket and symmetrically on the opposite side of the anterior chest. Ambient temperature was set at 66°F. Pacing modes during MRI were 0D0 for intrinsic rates > 40 and D00 for rates < 40. Diffusion scan sequences were excluded in PM dependent pts. Devices were St. Jude (n = 11), Boston Scientific (n = 12) or Medtronic (n = 11). The primary endpoint was change in device-side versus control-side skin temperature.

## Results

Results: Pt age was 61 ± 17 y; 23 were males. 28 PMs and 6 ICDs were scanned. 17 pts were PM dependent. Scan location was 16 head/neck/shoulder, 13 lumbar spine, 1 liver, and 2 lower extremity. Scan duration was 41.4 ± 14.95 min. At pre-scan baseline, device and control temperatures were 92.1 ± 1.8 and 91.0 ± 3.2°F, respectively (p = 0.09), and post-scan, 91.7 ± 3.2 and 90.1 ± 3.2° (p = 0.04). Temperature changes (post- vs. pre-scan) were -0.35 ± 2.8° (range, -8.0 to +4.8°) and -0.87 ± 3.9° (-11.8 to +7.4°), p = 0.56, and were similar for PMs and ICDs. No sensation of warmth was reported. No significant changes in lead or PG parameters were noted.

## Conclusions

PMs/ICDs cause mild local tissue/skin warming, but MRI did not cause additional, incremental heating. This suggests that heating of PG-can/local tissue with standard non-thoracic MRI scanning with selected contemporary devices appears to be generally mild or negligible. Results of a currently enrolling large registry study (MagnaSafe) will be required to firmly establish patient and device safety. Additional trials will be needed to assess PG heating and safety during thoracic MRI scans.

## Funding

None.

